# Disease-specific quality of life evaluation and its determinants in Cushing’s syndrome: what have we learnt?

**DOI:** 10.1007/s11102-013-0484-2

**Published:** 2013-04-07

**Authors:** X. Badia, E. Valassi, M. Roset, S. M. Webb

**Affiliations:** 1 Health Economics and Outcomes Research, IMS Health, C/Dr. Ferran 25-27, 2º, 08034 Barcelona, Spain; 2 Endocrinology/Medicine Departments, Hospital Sant Pau, IIB-Sant Pau, Centro de Investigación Biomédica en Red de Enfermedades Raras (CIBERER Unit 747), ISCIII, Universitat Autònoma de Barcelona (UAB), Barcelona, Spain

**Keywords:** Quality of life, Cushing syndrome, Treatment for Cushing’s syndrome, Factors related to HRQoL

## Abstract

Cushing’s syndrome (CS) has a considerable negative impact on patient health-related quality of life (HRQoL). Two disease-specific instruments (the CushingQoL and the Tuebingen CD-25 questionnaire) are now available to assess the impact of the disease and its treatment on HRQoL. The purpose of this review was to summarize the characteristics of the studies which have used these two instruments to date and summarize their findings regarding (a) the determinants of disease-specific HRQoL in patients with CS and (b) the impact of treatment for CS on disease-specific HRQoL. A total of 7 studies were identified, 5 with the CushingQoL and 2 with the Tuebingen CD-25. Most were observational studies, though the CushingQoL had been used in one randomized clinical trial. In terms of clinical factors, there was some evidence for an association between UFC levels and disease-specific HRQoL, though the presence and strength of the association varied between studies. There was also some evidence that a more recent diagnosis of CS could lead to poorer HRQoL, and that length of time with adrenal insufficiency may also affect HRQoL. There was no evidence for an impact on disease-specific HRQoL of etiology or of the clinical signs and symptoms associated with CS, such as bruising, rubor, and fat deposits. One factor which did have a significant negative effect on HRQoL was the presence of depression. No clear picture emerged as to the effect of demographic variables such as age and gender on HRQoL scores, though there was some evidence for poorer HRQoL in female patients. As regards treatment, the two interventions studied to date (transsphenoidal surgery and pasireotide) both showed significant gains in HRQoL, with moderate to large effect sizes. This type of review is useful in summarizing knowledge to date and suggesting future research directions.

## Introduction

Cushing’s syndrome (CS) is a rare endocrine disorder caused by chronic exposure to hypercortisolism, with an annual incidence of 2–3 cases per million [1]. It is most often due to a pituitary adenoma (Cushing’s disease) but may also be of adrenal origin (adenoma/s or carcinoma), or due to an ectopic secretion of adrenocorticotrophin (ACTH) by a tumor of any location.

The condition is more prevalent in females, who are at 3 times greater risk than males, especially in Cushing’s disease. Since CS leads to many physical problems, including central obesity, gonadal dysfunction, hirsutism, muscle weakness, hypertension, and hyperglycemia [2], as well as to psychiatric and psychological disturbances in the active hypercortisolemic state, such as major depression, mania, anxiety disorders, and cognitive impairment, the condition obviously has a profound effect on patients’ quality of life [3]. The negative influence on quality of life extends into areas such as body image, due to changes in physical appearance, as well as interference with family life and relations with partners, and impaired school or work performance [4]. Even after endocrine cure, patients score lower on measures of general well-being, anxiety and depression, and overall quality of life than healthy controls [5]. The assessment of health-related quality of life (HRQoL) is therefore of great relevance in patients with CS.

Health-related quality of life was initially assessed in CS patients using generic measures such as the SF-36 [6] and the SF-12 [7]. Measures of specific symptoms associated with the disease, such as the Hospital Anxiety and Depression Scale, were also used [8]. However, more recently, two disease-specific measures, the CushingQoL [9, 10] and the Tuebingen CD-25 [11, 12], have been developed. In principle, disease-specific measures of HRQoL should be able to tell us more about both the dimensions which are particularly relevant to CS patients as well as which factors influence HRQoL in CS patients. Given that these questionnaires have now been used in several studies, reviewing the results might help to provide a clearer picture of how CS and its treatment affect patients’ quality of life and potentially indicate directions for future research with this type of instrument.

The objective of this paper was to describe the studies performed to date using CS-specific HRQoL questionnaires and to summarize their findings. We were particularly interested in trying to establish what the determinants of disease-specific HRQoL in CS patients are and on understanding how interventions for CS impact it.

## Methods

This was a qualitative review of published studies for the two questionnaires of interest and additional available data for the CushingQoL. Published studies were identified from a review of the literature and from the authors’ own archives.

### The CushingQoL questionnaire

CushingQoL is a disease-specific questionnaire designed to assess HRQoL in CS. It is a self-administered instrument consisting of 12 questions which cover the areas of trouble sleeping, wound healing/bruising, irritability/mood swings/anger, self-confidence, physical changes, ability to participate in activities, interactions with friends and family, memory issues and future health concerns. Content for the questionnaire was derived from interviews with 10 patients with the condition [9]. Patients respond on Likert scales with five response categories (‘Always’, ‘Often’, ‘Sometimes’, ‘Rarely’, and ‘Never’, or ‘Very much’, ‘Quite a bit’, ‘Somewhat’, ‘Very little’, and ‘Not at all’). Responses are scored on a scale of 1–5, where ‘1’ corresponds to ‘Always’ or ‘Very much’ and ‘5’ to ‘Never’ or ‘Not at all’. The overall score is calculated by summing responses on all items and ranges from 12 (worst HRQoL) to 60 (best HRQoL). To facilitate the interpretation of scores, they can be standardized on a scale from 0 (worst HRQoL) to 100 (best HRQoL).

### The Tuebingen CD-25 questionnaire

The Tuebingen CD-25 questionnaire was also developed as a disease-specific questionnaire for patients with Cushing’s disease. Items in the questionnaire were generated from a review of the technical literature, interviews with patients, and the rating of neurosurgeons, endocrinologists and a neuropsychologist. The final version of the questionnaire consists of 25 items measuring the subdomains of Depression, Sexual Activity, Environment, Eating Behaviour, Bodily Restrictions, and Cognition. Response options are on a five-point Likert scale from 0 (strongly disagree), 1 (somehow agree), 2 (sometimes agree), 3 (mostly agree), and 4 (strongly agree). Each subscale and the total score have a minimum score of 0 and a maximum score of 100, with higher scores representing lower HRQoL.

### Studies using the CushingQoL and Tuebingen questionnaires

The two questionnaires have been used in a number of studies or registries, as described below and summarized in Table 1.

**Table 1:** Characteristics of studies using disease-specific questionnaires to assess quality of life in patients with Cushing’s syndrome

First author and year of publication	Questionnaire used	Type of study	Study design	Setting	Number of patients	Inclusion/exclusion criteria
Webb et al. [9]	CushingQoL	Validation study	Cross-sectional	5 European countries (Spain, France, Germany, the Netherlands, Italy)	125	Patients with histologically determined CS of pituitary or adrenal origin
Valassi et al. [15]	CushingQoL	European Registry on Cushing’s syndrome (ERCUSYN)	Cross-sectional, observational	23 European countries	508	Any etiology. Patients with adrenal cancer excluded.
Colao et al. [13]	CushingQoL	Clinical trial	Randomized, double-blind, phase 3 of pasireotide	11 European countries, Argentina, Brazil, Canada, USA, China and Turkey.	162	Confirmed persistent or recurrent Cushing’s disease or newly diagnosed disease, if they were not candidates for surgery.
Santos et al. [16]	CushingQoL	Study to evaluate CushingQoL test–retest reliability and sensitivity	Longitudinal study	Spain	59	None specified
Tiemensma et al. [17]	CushingQoL	Exploration of impact of illness perceptions on HRQoL	Observational Patients completed questionnaires at home	Netherlands	52	Age > 18 years and in remission for at least 1 year
Milian et al. [11, 12]	Tuebingen CD-25	Development and validation study	Cross-sectional, retrospective	Germany	63	Age > 18 years with pituitary-dependent Cushing’s disease.
Milian et al. [11, 12]	Tuebingen CD-25	Normative study	Cross-sectional	Germany	63 patients with CD 1784 healthy controls	None specified for control group

#### The CushingQoL validation study

This study [9] was performed in 5 European countries (Spain, France, Germany, the Netherlands, and Italy) between August and October 2006. CushingQoL was administered to 125 patients aged 18 years or above with histologically determined CS of pituitary or adrenal origin, or whose hypercortisolism disappeared after adrenal or pituitary surgery. Patients with CS due to adrenal carcinoma, ectopic ACTH secretion, or exogenous treatment with glucocorticoids were excluded. CushingQoL was self-administered at a single study visit. Other data collected included age, gender, level of education, current employment status, weight, height, blood pressure, date of diagnosis of CS and cause, history and persistence or not of adrenal insufficiency and hypercortisolism, surgery undergone for the disease, and history, dose, and date of pituitary radiotherapy, recent 24 h urinary free cortisol (UFC, within the last 6 months), current pharmacological treatment, concomitant diseases and their treatment, and hospital admissions over the last year related to CS or its complications.

#### Randomized phase 3 study of pasireotide in Cushing’s disease

This double-blind, phase 3 study tested the efficacy and safety of pasireotide, a somatostatin analog with high binding affinity for the somatostatin-receptor subtype 5. It included 162 adults with CS and urinary free cortisol at least 1.5 times the upper limit of the normal range who received subcutaneous pasireotide at a dose of 600 μg (82 patients) or 900 μg (80 patients) twice daily. Patients with urinary free cortisol not exceeding 2 times the upper limit of the normal range and not exceeding the baseline level at month 3 continued to receive their randomly assigned dose; all others received an additional 300 μg twice daily. The primary end point was urinary free cortisol at or below the upper limit of the normal range at month 6 without an increase in dose. Open-label treatment continued through month 12. Health-related quality of life was measured with the CushingQoL questionnaire. The main analysis focused on the impact of pasireotide on clinical markers [13] while further analyses examined the impact of the drug on HRQoL [14]. The data were also used to further examine the psychometric properties of CushingQoL.

#### The ERCUSYN registry

The European Registry on Cushing’s syndrome (ERCUSYN) [15] is a web-based, multicentre registry that enrolled 508 CS patients diagnosed between January 1st, 2000 and October 31st, 2010 from 36 centers in 23 European countries. The registry used a mixture of prospective and retrospective recruitment, though primarily prospective (83 % from 2008 onwards). Patients were classified, depending on the diagnosis, into pituitary-dependent CS (PIT-CS), adrenal-dependent CS (ADR-CS), CS from an ectopic source (ECT-CS), and CS from other etiologies (OTH-CS). Patients with adrenal cancer were excluded. Data in the ERCUSYN database included baseline demographic and anthropometric characteristics, etiology of CS and diagnosis date, comorbidities, bone status, and CS therapy, among others. Health-related quality of life was measured using the CushingQoL and EQ-5D generic questionnaire. Long-term treatment outcomes were assessed using biochemistry and imaging parameters, post-treatment hormone replacement therapies, pituitary deficiencies, clinical features, HRQoL, and bone status. Urine 24 h free cortisol (UFC) levels were collected and assessed to determine whether they were within the range of normal values.

Additional analysis was performed using data from the ERCUSYN study for the current manuscript to analyze the relationship between CushingQoL scores and clinical variables and to assess the impact of transsphenoidal surgery (TSS) on HRQoL.

#### Psychometric performance of the CushingQoL questionnaire in conditions of real clinical practice

In this study [16], the authors aimed to evaluate the psychometric properties of test–retest reliability and sensitivity to change of the CushingQoL questionnaire under conditions of usual clinical practice conditions in a tertiary referral centre. To evaluate test–retest reliability, the CushingQoL was administered twice to ‘cured’ patients, and to patients with active hypercortisolism (“active group”). Patients were considered cured if they had demonstrated adrenal insufficiency or morning cortisol suppression [<50 nmol/L] after 1 mg dexamethasone overnight, and if repeated measurement showed normal (<280 nmol/L) values for 24 h urinary free cortisol. To evaluate sensitivity to change, the questionnaire was administered to patients with active hypercortisolism at baseline, on diagnosis of CS, and again at 4 ± 1.5 and 9 ± 3 months after successful surgery.

#### Impact of negative illness perceptions on HRQoL after long-term remission of CS

In this study [17], 52 patients completed questionnaires including the Illness Perception Questionnaire (IPQ)-Revised, and CushingQoL to “explore illness perceptions, as potentially modifiable psychological factors, in relation to HRQoL in patients with long-term remission of CS”. Patients completed the questionnaires at home. Results were compared with reference data from recent studies including patients with vestibular schwannoma (n = 80), acute (n = 35) or chronic (n = 63) pain, and chronic obstructive pulmonary disease (COPD; n = 171).

#### The development of the Tuebingen Cushing’s disease quality of life inventory (Tuebingen CD-25). Part I: construction and psychometric properties

As the title indicates, this paper reports the procedures used to develop and validate the Tuebingen CD-25. The validation part of the study was carried out in a sample of 63 patients with an established diagnosis of pituitary-dependent CD, of whom 13 were recruited after admission for operative treatment, 15 were newly diagnosed, as yet untreated patients, and 35 had been operated on between 3 months and 9 years before participating in the validation study [11]. Patients completed the items that would eventually constitute the Tuebingen CD-25 together with additional potential items for the questionnaire which were later discarded. They also completed the WHOQoL-BREF [18] and the effect of hormone levels (preoperative ACTH, serum cortisol levels, 24 h UFC) and age on Tuebingen CD-25 scores were analyzed.

#### The development of the Tuebingen Cushing’s disease quality of life inventory (Tuebingen CD-25). Part II: normative data from 1,784 healthy people

The second phase in the development of the Tuebingen CD-25 consisted of obtaining normative data from a healthy control group (n = 1784) which could be compared with the results obtained from the 63 CD patients. The healthy control group was recruited via a German online internet version of the questionnaire and additional information about age, gender, and educational background was also obtained. The healthy control group included 1,210 women and 574 men with a mean age of 28.9 years [12].

## Results

The main results of the review of the different studies using disease-specific measures to assess HRQoL in patients with CS are shown in Table 2.

**Table 2:** Main findings of studies reviewed

Study	Main findings
Webb et al. [9]	Significant differences on CushingQoL scores between patients with active [mean 46.1 (SD 22.6)] and controlled [mean 56.1 (SD 19.5)] disease (based on UFC levels) (*P* = 0.009) Significant differences between patients with [mean 47.6 (SD 20.5)] and without [mean 56.1 (SD 22.3)] hypercortisolism (*P* = 0.043) Lower (poorer) CushingQoL scores in patients with a more recent diagnosis (last 2 years; mean score 44 ± 22) than in those diagnosed earlier (mean score 59 ± 22; *P* < 0.001) Linear regression analysis showed that being female (*P* = 0.029) and having elevated UFC (*P* = 0.011) were the main contributors to impaired HRQoL
Colao et al. [13]	Differences in CushingQoL scores between patients with fully controlled mean UFC [58.0 (3.7)] and those with uncontrolled UFC [51.5 (3.4)] were not statistically significant No statistically significant differences were observed between groups classified by BMI, waist circumference, weight, amount of facial rubor, striae, bruising, supraclavicular fat, dorsal fat, or Karnofsky scores Consistent and significant differences related to depression. Mean CushingQoL scores in patients with minimal depression [mean = 61.6 (SE = 2.2) at month 12] were significantly higher (better HRQoL) than for patients with severe levels of depression based on the BDI-II [mean = 30.8 (SE = 6.9) at month 12] A high percentage of patients showed a significant improvement in terms of HRQoL (with improvements over 10 points on CushingQoL) at 3 months (50.0 % of patients), 6 months (48.0 %) and 12 months (52.0 %) CushingQoL score improved by 11.1 points (95 % CI, 6.8–15.5) after 12 months of treatment with pasireotide, representing effect sizes of 0.47 and 0.49 (for 600 ug)
Valassi et al. [15]	No significant differences in CushingQoL score by etiology In multiple regression analysis, only depression was found to independently predict lower CushingQoL scores Transsphenoidal surgery produced improvements on CushingQoL from a mean (SD) score of 38.3 (22.1) at baseline to 56.6 after 12 months (a change of 18.3 points), representing an effect size of 0.82
Santos et al. [16]	CushingQoL scores were worse in patients with active disease than in those defined as ‘cured’ (46 ± 15 vs. 58 ± 20, *P* < 0.05) No differences were seen between males and females with active disease nor between patients with hypercortisolism of adrenal and pituitary origin No differences in score between patients who had undergone radiotherapy and those who had not No statistically significant correlation was found between CushingQoL scores and age Transsphenoidal surgery produced improvements on CushingQoL from a mean (SD) of 41 (15) at baseline to 54 (13) at 4 months (*P* < 0.01) and 68 (17) at 9 ± 3 months post surgery (*P* < 0.001), corresponding to effect sizes of approximately 0.86 and 1.8, respectively. Sample size was small (n = 11)
Tiemensma et al. [17]	Negative illness perceptions were associated with poorer HRQoL
Milian et al. [11, 12]	Non-linear correlation between Tuebingen CD-25 scores and patients’ age Preoperative 24 h urinary free cortisol (UFC) levels correlated significantly with the subscale Cognition and only marginally failed significance level for the subscale Eating Behaviour Preoperative cortisol and ACTH levels did not correlate with any scale
Milian et al. [11, 12]	Female patients perceived worse HRQoL than men in the domains of depressive symptoms and social environment (*P* < 0.05)

### Determinants of disease-specific HRQoL

In the original validation study [9], significant differences were observed in CushingQoL scores between those with active [mean 46.1 (SD 22.6)] and controlled [mean 56.1 (SD 19.5)] disease (based on UFC levels) (*P* = 0.009) and between patients with [mean 47.6 (SD 20.5)] and without [mean 56.1 (SD 22.3)] hypercortisolism (*P* = 0.043). The authors also found poorer CushingQoL scores in patients with a more recent diagnosis (last 2 years; mean score 44 ± 22) than in those diagnosed earlier (mean score 59 ± 22; *P* < 0.001). In pituitary-dependent CD, they found no differences between those with and without hypopituitarism or between those who had prior pituitary radiotherapy and those who had not. Linear regression analysis showed that being female (*P* = 0.029) and having elevated UFC (*P* = 0.011) were the main contributors to impaired HRQoL, while time elapsed since diagnosis of adrenal insufficiency was slightly but positively related with the CushingQoL score (r = 0.35, *P* = 0.02); in other words, the longer the duration of adrenal insufficiency, the greater the impact on HRQoL. Current age and age at diagnosis were not significantly correlated with CushingQoL scores and no correlation was observed between time elapsed since surgery and CushingQoL score nor between CushingQoL score and the presence of adrenal insufficiency.

Analysis of data from the pasireotide clinical trial showed a clear trend, after 12 months of treatment, to differences in CushingQoL scores between patients with fully controlled mean UFC [58.0 (3.7)] and those with uncontrolled UFC [51.5 (3.4)], though the differences were not statistically significant. Likewise, no statistically significant differences were observed between groups classified by BMI, waist circumference, weight, amount of facial rubor, striae, bruising, supraclavicular fat, dorsal fat, or Karnofsky scores. In the same study, consistent and significant differences were found at month 6 and month 12 related to depression. Specifically, CushingQoL mean scores in patients with minimal depression [mean = 61.6 (SE = 2.2) at month 12] were significantly higher than for patients with severe levels of depression based on the BDI-II [mean = 30.8 (SE = 6.9) at month 12].

In the ERCUSYN registry, no significant differences in CushingQoL score were observed between patients with pituitary dependent CD (mean score 40 ± 17), adrenal dependent CS (mean score 39 ± 14), CS from an ectopic source (mean score 27 ± 13), or those with other types of CS (mean score 23 ± 2) studied at baseline. CushingQoL data were only available in the ectopic and other group from n = 6 and n = 2 patients, respectively. Multiple regression analysis was carried out with ERCUSYN data in which gender, age, diagnosis, delay to diagnosis, BMI, depression, diabetes, and hypertension were independent variables, and CushingQoL score the dependent variable. Depression was the only variable found to independently predict lower CushingQoL scores.

In the Santos et al. study [16], CushingQoL scores were observed to be worse in patients with active disease than in those defined as ‘cured’ (46 ± 15 vs. 58 ± 20, *P* < 0.05). No differences were seen between males and females with active disease nor between patients with hypercortisolism of adrenal and pituitary origin (although the authors reported a trend for lower CushingQoL scores in patients of adrenal origin), or between patients who had undergone radiotherapy and those who had not. No statistically significant correlation was found between CushingQoL scores and age.

In the study by Tiemensma et al. [17], the authors found moderate to strong inverse correlations between results on the different dimensions of the IPQ-R questionnaire and the CushingQoL indicating that more negative illness perceptions were associated with poorer HRQoL. This was particularly true for the dimensions of ‘Illness identity’ (r = 0.659; *P* < 0.01) and emotional representation (r = 0.629; *P* < 0.01).

In the Tuebingen development study [11], the authors found a non-linear correlation between the Tuebingen CD-25 scores and patients’ age; younger (21–30 years) and middle-aged (51–60 years) patients had poorer HRQoL than patients between 31 and 50 years and those over 61 years of age. They also found that preoperative 24 h urinary free cortisol (UFC) levels correlated significantly with the subscale Cognition and only marginally failed significance level for the subscale Eating Behaviour, while preoperative cortisol and ACTH levels did not correlate with any scale. In the Tuebingen normative study, the authors observed that female patients perceived a worse HRQoL than men in the domains depressive symptoms and social environment (*P* < 0.05).

### Impact of interventions for CS, and changes in HRQoL over time

Three of the studies reviewed [13, 15, 16] included repeated measurements with CushingQoL and examined the impact of different interventions on patients’ disease-specific HRQoL. To date, no longitudinal studies have been performed with the Tuebingen CD-25 questionnaire.

In the pasireotide study, CushingQoL score improved by 11.1 points (95 % CI, 6.8–15.5) after 12 months of treatment with the study drug [13]. Effect sizes for changes at 6–12 months were 0.57 and 0.57 for 900 ug and 0.47 and 0.49 for 600 ug, respectively, which would represent moderate effect sizes [14, 19]. Degree of control of UFC was also found to be important, with clinically meaningful improvements (i.e. an improvement of more than 10.1 points of the CushingQoL score) observed in patients whose UFC levels were controlled or partially controlled at month 6. A high percentage of patients showed a significant improvement in terms of HRQoL (with improvements over 10 points on CushingQoL) at 3 months (50.0 % of patients), 6 months (48.0 %) and 12 months (52.0 %). On the other hand, there were no clinically meaningful improvements in the group with uncontrolled UFC.

Data from the ERCUSYN registry showed that transsphenoidal surgery (TSS) produced improvements on CushingQoL from a mean (SD) score of 38.3 (22.1) at baseline to 56.6 after 12 months (a change of 18.3 points), corresponding to an effect size of 0.82 (large effect size). The Santos et al. [16] study showed similar results for TSS, with CushingQoL scores improving from a mean (SD) of 41 (15) at baseline to 54 (13) at 4 months (*P* < 0.01) and 68 (17) at 9 ± 3 months post surgery (*P* < 0.001). In this case too, the differences correspond to effect sizes of approximately 0.86 and 1.8, respectively, although the sample size used for this analysis was small (n = 11).

In terms of correlations in change scores, in the pasireotide study the authors found a moderate correlation of −0.40 (*P* < 0.01) between changes in the CushingQoL score and changes in mean UFC from baseline to month 12, with increasing CushingQoL scores (improved HRQoL) as mean UFC levels declined. Statistically significant correlations (*P* < 0.01) were also observed between changes in CushingQoL score and changes in BMI (r = −0.31), weight (r = −0.32), and BDI-II (r = −0.59). Only small or null correlations were observed between changes in CushingQoL scores and sitting systolic blood pressure, waist circumference, facial rubor, striae, brusing, supraclavicular fat pad, and dorsal fat pad (though there were some differences between the 600 and 900 ug drug dose groups). Furthermore, based on a minimally important difference (MID, the smallest clinically relevant difference in CushingQoL score) of 10.1 [20], there was a clinically meaningful improvement in CushingQoL scores at month 6 in patients whose UFC levels were controlled (n = 27; mean improvement 12.8; SD = 15.1) or partially controlled at month 6 (n = 17; mean improvement: 10.7; SD = 20.8), but the improvement in CushingQoL scores did not quite reach the MID in the uncontrolled group (n = 29; mean improvement: 9.9; SD = 21.8). Interestingly, in additional analyses carried out with data from the pasireotide study [14] it was found that of patients who completed 12 months of therapy in that trial, 80 % had either a reduction in diastolic blood pressure ≥5 mmHg (58 %), a decrease in weight ≥7 % (42 %), or an improvement ≥MID on CushingQoL (46 %) (mean ± SD, 11.1 ± 15.2). Improvements in symptoms and HRQoL were maintained at 12 months.

## Discussion

This review of studies which have evaluated HRQoL in CS using disease-specific questionnaires has indicated a number of findings which may help to understand the relationship between the clinical features of Cushing’s syndrome, its treatment, and HRQoL.

In terms of clinical factors, there is some evidence for an association between UFC levels and disease-specific HRQoL, though the strength of the association varied between studies. There was also some evidence that a more recent diagnosis of CS could lead to poorer HRQoL, and that length of time with adrenal insufficiency (though not the presence of the condition) may also affect HRQoL. On the other hand, there is no evidence to date for an impact of etiology or of the clinical signs and symptoms associated with CS, such as bruising, rubor, and fat deposits, on disease-specific HRQoL. This latter finding may be somewhat surprising, though the apparent lack of effect may have been due to the low numbers of patients with moderate to severe levels of those symptoms, in the one study which examined this aspect. One factor which did have a significant negative effect on HRQoL was the presence of depression. This was clearly seen in two of the studies reviewed.

No clear picture emerged as to the effect of demographic variables such as age and gender on HRQoL scores, though there was some evidence in two of the studies that female patients scored lower than males on CushingQoL and on some of the Tuebingen CD-25 dimensions. Poorer HRQoL in females is also consistent with the findings of other studies both in patients [21, 22] and the general population [23]. Finally, as regards treatment, the two interventions studied to date (TSS and pasireotide) both showed significant gains in HRQoL, with moderate to large effect sizes.

Obviously, the findings of the present review should be taken as provisional given the small number of studies and the characteristics of some of the studies, such as the low number of patients in some sub-groups. It should also be borne in mind that the association between some clinical variables, such as UFC levels and HRQoL can be obscured by the fact that even those patients who have achieved a long-term cure can still report decreased HRQoL, with physical and psychosocial impairments, especially in the presence of hypopituitarism [5]. Furthermore, variations are known to exist in UFC measurements both methodologically and physiologically on a day-to-day basis [13].

Regarding the effect of treatment, it is interesting to note that changes observed with pasireotide from baseline to 6–12 months were comparable to the differences observed between patients with active and non-active disease (differences of approximately 10–11 points on CushingQoL). Similarly, although treatment with pasireotide showed slightly lower changes than those obtained with TSS, CushingQoL score at 6 months in patients treated with the drug (53.5) was similar to that seen in patients 6 months after receiving TSS (54.4). Based on their CushingQol scores, patients in the surgical group started from a lower baseline HRQoL than those treated with pasireotide.

In relation to the measurement of change over time with these instruments, it is of note that earlier studies suggested a ‘ceiling’ for HRQoL in CS patients, at least using a generic HRQoL instrument [24]. It would be interesting to determine whether a similar effect exists with disease-specific instruments, as may be suggested by the fact that both pasireotide and TSS appeared to achieve similar CushingQoL scores after 6 months. In that sense, the initiative by the developers of the Tuebingen CD-25 to obtain normative scores from patients and healthy controls is positive [12]. It would be useful to have such normative scores for CushingQoL as well, if possible, as they provide clinicians and researchers with a reference point or benchmark for evaluating their own populations, and can help determine what might be realistic upper levels of HRQoL. Other avenues for future research include a closer investigation of the relationship between CS signs and symptoms such as bruising, rubor, and striae, and HRQoL. The lack of evidence for an effect in the present review may have been due to the low numbers of patients with moderate to severe levels of the symptoms, but that should be confirmed in future studies with larger numbers of patients. Future studies could also attempt to clarify whether female patients do, in fact, have poorer HRQoL when measured using disease-specific instruments and, if so, to try to identify the dimensions affected, as that can indicate areas to which interventions could be targeted.

Finally, we noted that the presence of depression quite consistently showed a negative effect on CushingQoL scores. This is perhaps not surprising, given previous evidence showing the impact of depressive symptoms on HRQoL in a range of pathologies and conditions [25–27]. Clearly, this is a factor to be taken into account both when planning studies and analyzing their results, and when deciding on management of CS patients. Studies using HRQoL as an endpoint should consider including a measure of depression such as the BDI-II to be able to control for this potentially confounding factor in analyses. Future studies could also examine whether interventions to treat the depression have a positive impact on disease-specific HRQoL in CS patients.

In conclusion, we have reviewed studies and other applications of HRQoL questionnaires in CS patients to try to clarify what are the determinants of HRQoL in these patients and how treatment impacts results on HRQoL questionnaires specific to the disease. There appears to be some evidence that elevated, uncontrolled levels of UFC are associated with poorer HRQoL, and that improving UFC control leads to better HRQoL, and also that depression is an important factor for poorer HRQoL in these patients. On the other hand, there is currently no or only weak evidence for an impact of other signs and symptoms, and etiology on disease-specific HRQoL. Further studies are required into these areas, and the importance of having normative, reference scores for disease-specific questionnaires has been noted. This type of review is useful in summarizing knowledge gained with these questionnaires to date and suggesting future avenues of research.
